# Synthesis and characterization of new organometallic lanthanides metal complexes for photodynamic therapy

**DOI:** 10.1038/s41598-024-75800-8

**Published:** 2024-10-30

**Authors:** H. A. Fetouh, E. H. El-Mossalamy, J. M. El Desouky, Mervette El Batouti

**Affiliations:** 1 Chemistry Department, Faculty of Science, Alexandria University, Alexandria, Egypt; 2 Chemistry Department, Faculty of Science, Benha University, Banha, Egypt; 3 Clinical Pathology Department, Faculty of Medicine, Alexandria University, Alexandria, Egypt

**Keywords:** Photodynamic activity, Ligand, Metal, Complex, Photosensitizer, Schiff bases, Biochemistry, Cancer, Chemical biology, Medical research, Chemistry, Materials science

## Abstract

New Schiff base ligand: 4-methoxy salicaldhyde-2-2-phenyl-hydrazono acetaldehíyde prepared by facile method. The molecular structures characterized by elemental analysis and proton magnetic resonance spectra (^1^H-NMR spectra). This spectra at the chemical shifts (3.5–10.39 ppm) confirmed the types and the numbers of protons. The sharp melting point at the range 110–112 °C confirmed purity. New optically active metal (samarium, terbium and gadolinium) complexes of the Schiff base synthesized in a one pot reaction. Vibrational IR spectra confirmed functional groups. Scanning electron microscopy micrographs confirmed that the modified microstructure of the metal complexes differed in morphology than the ligand. Powder X-ray diffraction patterns confirmed good crystalline structure. The optically activity of the solid metal complexes confirmed from electronic absorption spectra. The UV absorbance band at the wavelength range 280–390 nm and the intense phosphorescence bands up to 830 nm enabled application in photo dynamic therapy for apoptosis cancer cells by conversion triplet oxygen in the tissues into reactive singlet oxygen. Low charge transfer energy: 2.59–2.61 eV, high molar extinction coefficients (ε) at the order of magnitude $\:{10}^{6}$ M^− 1^ cm^− 1^ and the intense phosphorescence bands reflected good photodynamic activity. The metal complexes are thermally stable.

## Introduction

Schiff bases contain azomethine imines group are widely applicable^1^. Metal Schiff bases complexes are applied in the organic synthesis, as conductive solid electrolytes, in host-guest supra molecular chemistry, sensitive sensors, medicine and catalysis^2^.Transition metal complexes of tetra dentate Schiff bases ligands are electrochemically active catalysts^3^. Bis-hydrazones metal complexes have potent biological activity^4^. Bis-hydrazones, glyoxal, biacetyl, benzyl, 2,4-di-hydroxy-bebzaldhyde and 4-methoxy salicaldhyde are important multi dentate ligands for several metal ions^5^. These new colored materials required in all fields of technologies such as energy storage super capacitor electrodes and photodynamic therapy (PDT)^6,7^.

PDT uses optically active photosensitizer (S) therapeutic drug (nontoxic in dark conditions) injected to the patients and diffuses inside the cells. The molecules excited by absorption laser photon energy (h$\:\upsilon\:)$ just sufficient for excitation not damage normal tissue. Excited molecule rapidly undergo non-radioactive efficient intersystem crossing with high quantum yield of triplet state interact with inert triplet molecular oxygen (^3^O_2_) in the tumor cells giving energetic cytotoxic singlet oxygen (^1^O_2_) rapidly damage tumor cells and relaxed to triplet oxygen^6,8,9^.

According to Jablonski diagram: sensitizer absorbs light photon, relax via fluorescence and phosphorescence^10^. The long relaxation time (τ) allowed interaction with oxygen. Electron excited into higher-energy excited singlet state. Energy loss by rapid decay through vibrational and rotational sublevels via internal conversion populate excited singlet states. Radiative fluorescence decay lifetimes (τ_F_ 10^− 9^–10^− 6^ s) is spin allowed transitions and conserve electron spin multiplicity. Spin inversion of excited singlet states populates the lower-energy first excited triplet state via (ISC) transition followed by slow phosphorescence (τ_P_ 0.001–1.0 s)^11^. Drug sensitizer is safe to the target tissue until irradiation and slightly cytotoxic on excitation; preferential bio accumulates in diseased target tissue and rapidly cleared from the human body post treatment.

Certain skin cancer cells resist cell apoptosis by the protective singlet oxygen histidine amino acid scavenger. Photodynamic efficiency depend on life time and quantum yield of triplet state^11^. These values for metal phthalocyanines influenced by metal nature. Diamagnetic metals Zn, Al and Ga complexes 40% quantum yield and short life time Amide Zn-, Si(IV) nano composites with two trans axial ligands minimize aggregation such as siloxanes-2-methoxy ethylene-glycol are active. Conjugation improved photodynamic activity of lanthanide ions complexes. Diamagnetic complexes Lu(III) texaphyrins are effective in contrast to gadolinium complex. Life time and quantum yield of triplet state and ISC rate of diamagnetic complexes depend on the atomic number metal ion. Diamagnetic ions (Y, In, Lu) promoted ISC and elongated τ_T_ (187 −35 µs) favored than paramagnetic complexes (except Copper octa-ethyl benzochlorins) decreases life time of excited state and prevented photodynamic reactions^11^.

Zn and toxic Cd derivatives displayed unit triplet quantum yield. Sn(IV) purpurins more active than Zn(II) analogous. The photodynamic activity of diamagnetic complexes of sulphonated benzochlorins and porphyrins depends on the atomic number of the metal ion. High ISC rate of diamagnetic lanthanides complexes is due to the heavy atom effect. Schiff bases complexes of lanthanides samarium, terbium and gadolinium rarely reported^9,12^.

Many reported metals complexes have not evaluated for photodynamic therapy. Some lanthanides metal complexes improved fertility in male rats^13^. Tellurium 2-hydroxy‐1‐naphthaldehyde complexes are antioxidant and anti-tumor^14^.Anticancer nickel(II) hydrazone complexes evaluated in vitro and by molecular docking^15^. La(III), Y(III) N,N′-phenylene (bis1-cyclopropyl-7-(4-ethylpiperazin-1-yl)-6-fluoro-1,4-dihydroquinoline-3-carboxylic acid enhanced apoptosis of colon cancer cell by overexpression P53 protein^16^. Yttrium, lanthanum, zirconium and uranium complexes are antiviral agents against human cytomegalovirus^17^. Divalent ion Co, Ni and Cu complexes of ρ‐dimethylaminobenzaldehyde thiosemicarbazone reported as antitumor based on in-vitro evaluation and 50% inhibitive concentration^18^.

Antitumor: (quinidine nucleus Co(II), Ni(II), Zr(IV) nano-sized metal chelates complexes: 3‐((2‐methyl-quinolin‐4‐yl) diazenyl)naphthalen‐2‐ol HL^19^; Antitumor antibacterial aminothiazoles Co, Cu, Zn and Th complexes^20^; nano-metal complexes of sulfamerazine azo dye ^21^; Divalent metal ions complexes Cu, Ni, Zn, Cd, Hg, Zr and Ce sulfamerazine-resorcinol azo-dye are antitumor^22^; also Cr(III) pyrazolin derivatives^23^ and VO(II)‐thaizole complexes; Cu(II)-benzohydrazide nanometer complexes, anti-inflammatory and anti-allergic^24^; Zn(II) benzohydrazide improved traditional ammonium nitrate fertilizer^25^. None of these studies investigated photo physical processes of metal complexes. So this current study aims preparation of characterization of new Schiff base ligands and complexes of lanthanide metal ions and evaluation the photodynamic activities.

## Materials and methods

### Chemicals

Certified chemicals of analytical grades and high purity (4-methoxy salicaldhyde, 2-(2-phenylhydrazono) acetaldehyde and absolute ethanol) purchased from Sigma Aldrich Co. All these chemical used as received without further purification.

### Synthesis and characterization of the new hydrazone ligand

#### Synthesis of the ligand

A mixture of 1:1 molar ratio of 4-methoxy salicaldhyde “0.5 g” and (2-(2-phenylhydrazono) acetaldehyde) (0.1 mol; 0.086 g) dissolve in 50 mL absolute alcohol solvent containing few drops of concentrated hydrochloric acid and refluxed for 4 h. The product ligand: 4-methoxy salicaldhyde-2-2-phenyl-hydrazono acetaldehyde separated by solvent evaporation and recrystallized from ethanol.

#### Characterization of the ligand

The melting point uncorrected and taken in open capillary tubes using electrothermal apparatus 9100 (UK). The purity detected by the sharp melting point range (110–112 °C). The microanalyses operated at Faculty of Science, Cairo University, Cairo, Egypt, using Element Vario el III C, H, N, analyzer (Germany). ^1^H-NMR spectra in (CD_3_)_2_SO recorded on Varian FT-200 MHz spectrometer (400 MHz, *DMSO*-d_6_) using TMS as internal standard. Surface analysis (sample sputter coated with gold using scanning electron microscope JEOL SEM-5410 (Japan), 20 kV accelerating^26^. IR recorded using potassium bromide method on a Perkin-Elmer 1650 spectrophotometer (calibrated by polystyrene film), model 1430 at frequency 200–4000 cm^− 1^^27^.

Methoxy (OCH_3_) substituent group affected ligand structure by both inductive electron withdrawing group by inductive effect and predominant mesmeric effect as electron releasing group (nucleophile). Electron withdrawal group prevents aggregation of ligand molecules due to hydrophilic (O) that improved solubility and hydrophobic (CH_3_). The hydrophilic-hydrophobic nature of OCH_3_ improved stability of the ligand^28^.

### Synthesis of the new Hydrazone lanthanides metal complexes

**Fig. 1:**
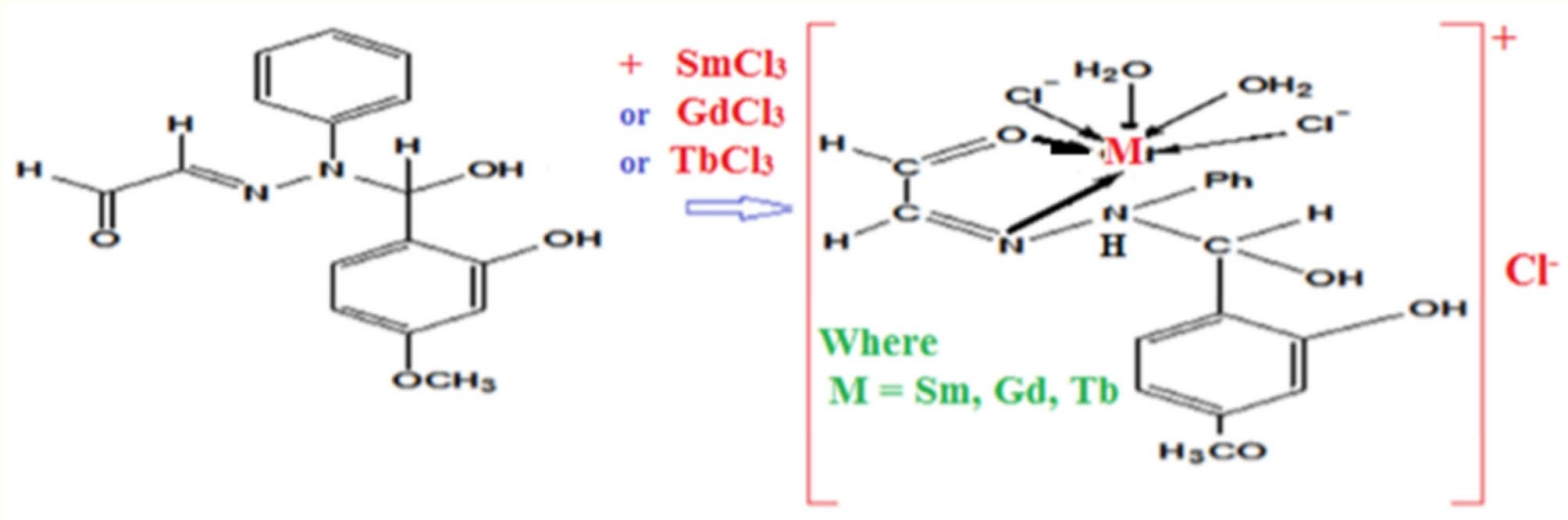
Suggested binding mode in lanthanides metal complexes.

The solid chelates of stoichiometric ratios (1:1) (M:L) for some selected metal were prepared by mixing the six hydrated metal chlorides (TbCl_3_, GdCl_3_ and SmCl_3_) with hot ethanolic solution of 4-methoxy salicaldhyde-2-2-phenyl-hydrazono acetaldehyde ligand, reflux 9.0 h; cooling to the room temperature. The solid chelates separated and recrystallized from absolute ethanol solvent, dried and preserved against atmospheric conditions in a desiccator over anhydrous calcium chloride^26^. The obtained metals complexes are poly crystalline and the chemical structure suggested based on the elemental analysis. The bonding mode represented in Fig. 1. These three new metal ions selected in this current study because high oxidation tendency improved complexation to the prepared ligand. These metals ions contain vacant outer d-orbitals can easily accept lone pairs of free electrons from the heteroatoms of the ligand forming strong covalent coordinate bond That can be reinforced by back donation of metal ligand charge transfer^27^.

**Fig. 2:**
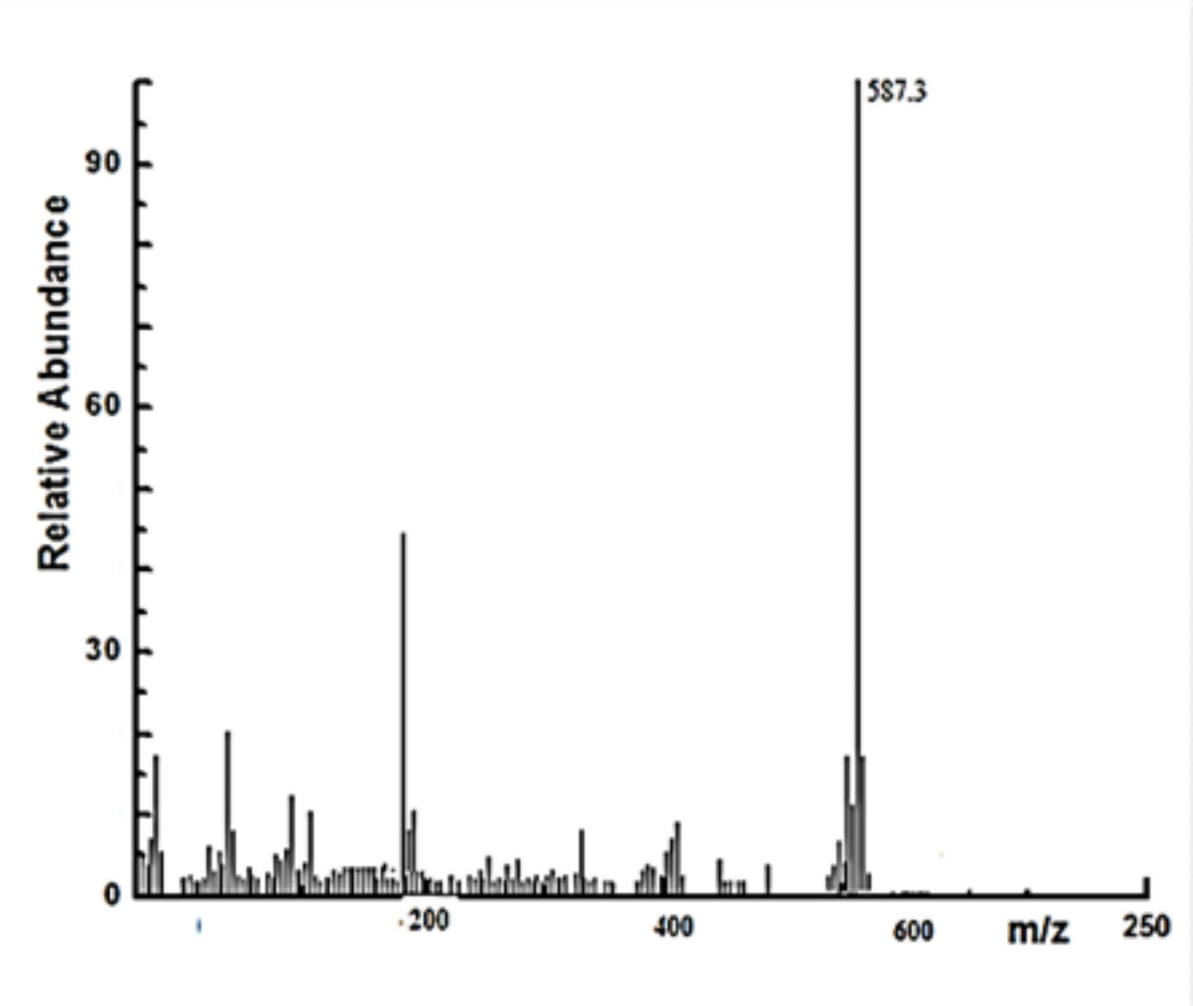
Mass spectra of Schiff base Gd(III) benzyl bis-hydrazone complex.

This binding mode confirmed by determination the observed molecular weight (Fig. 2) obtained from mass spectra (587.3 g mol^− 1^).This molecular weigh (Mw.) used in trials proposal the strucuture of the metal complex (Fig. 1) for which the calculated Mw. (573.7 g mol^− 1^). The good agreement between observed and calculated Mw. confirmed the suggested bonding mode is correct for Gd(III) complex^27^.

### Instrumental analysis for characterization of the ligand and metal complexes

The elemental analysis C, H, N and Cl contents performed using FlashSmart™ Elemental Analyzer. The percentage of metal ion content determined as: The complex digested and decomposed with aqua regia (3:1 volume per volume concentrated HCl to concentrated nitric acid. The free metal contents determined using Shimadzu -atomic absorption spectrophotometer, model 6650. Molecular structure of metal complexes confirmed by: FTIR (KBr) spectra by using Perkin Elmer spectrophotometer, Model 1430 at frequency range 200–4000 cm^− 1^ calibrated by polystyrene film; pXRD (at 25 °C, Bruker D8 advance XR Germany diffractometer contained Cu-anticathode Target (Cu-Kα radiation (λ 1.54060 Å at 40 kV) at incidence reflection angles (2θ°) 5–70°, 0.02° step and 1° min^−1^scan rate^27^.Optical properties investigated by UV-Vis. spectroscopy recording electronic absorbance spectra. Photo luminescence emission spectra in absolute ethanol recorded at ambient temperature from lowest-lying excited triplet metal ligand charge transfer to ground state recorded at the excitation wavelength 650 nm^28^. TGA characterized decomposition and thermal stability recorded by Perkin-Elmer TGA-7. Mass spectra recorded heating rate 15 ^°^C⋅min^− 1^ from RT to 800 °C in nitrogen atmosphere. Particle size distribution determined based on light scattering of metal complex suspension using NanoZS/ZEN3600 Zetasizer (Malvern, Instruments Ltd, Malvern, UK)^27–29^.

## Results and discussion

### Characterization of the ligand and metal complexes

Table 1 showed elemental CHN analysis data and tentative formula of the brown color Schiff base ligand and metal complexes.

**Table 1:** Elemental analysis of ligand.

Empirical formula	Molecular wt. (g mol^− 1^)	% Microanalysis					m.p. °C
		Metal (M)	C	H	*N*	Cl	
C_16_H_16_N_2_O_4_	300	–	67.61	5.92	12.48	–	110–112
SmC_16_H_16_N_2_O_4_.2H_2_O.2Cl	628.51	34.73	34.93	2.75	5.45	7.60	Above 300 °C
GdC_16_H_16_N_2_O_4_.2H_2_O.2Cl	635.40	34.89	35.11	2.88	5.60	7.75	
TbC_16_H_16_N_2_O_4_.2H_2_O.2Cl	637.08	27.87	37.20	3.52	6.39	13.51	

**Fig. 3:**
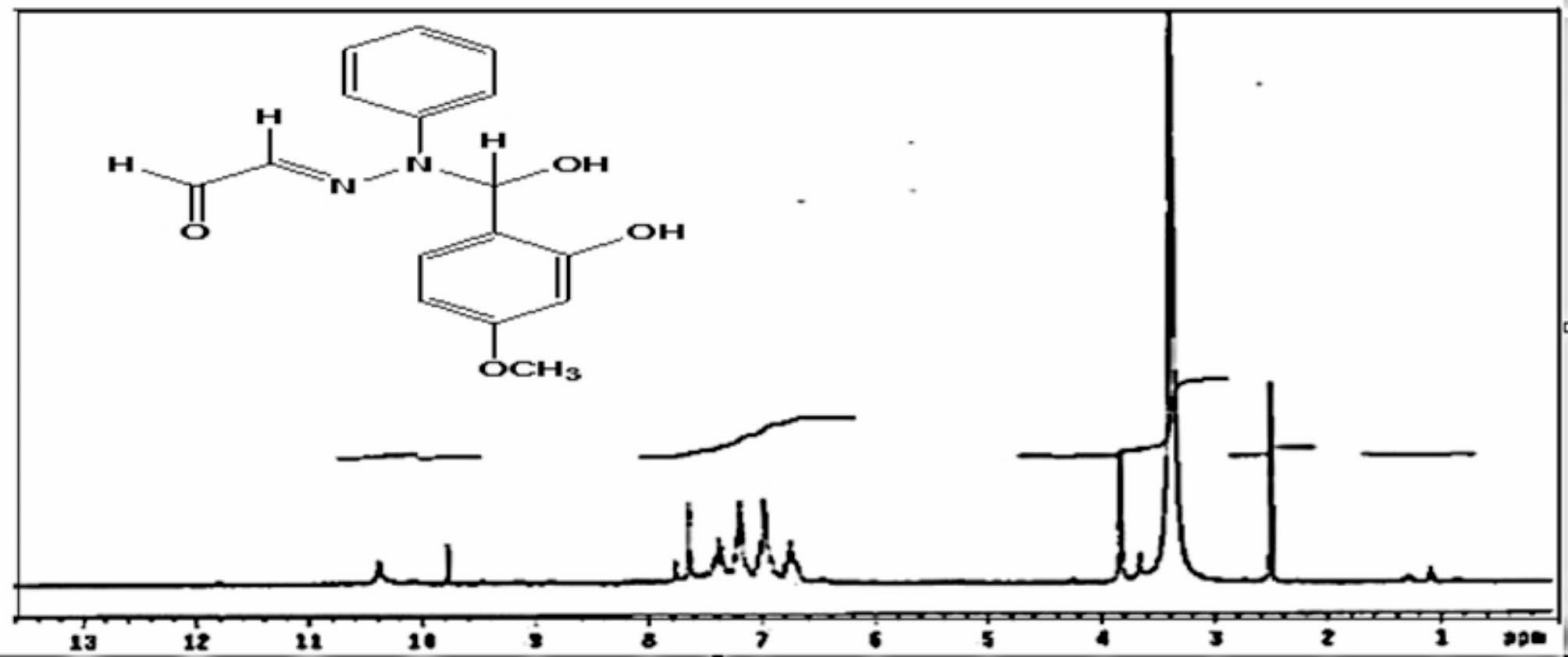
^1^H NMR of Schiff base ligand.

Agreement between observed and calculated atomic wt% confirmed suggested molecular structure. Relatively high m.p. confirmed stability of Schiff base ligand. Brown color suggested good Schiff base contains optically active chromospheres can interact with light photon. The inspection of ^1^H-NMR spectrum, Fig. 3 showed H of -CH = N- azomethine group appeared as a singlet signal at Chemical shifts (δ; ppm) range (3.5–10.4 ppm)^26,28^, Table 2.

The ^1^H-NMR signals of the different types of protons expected for the new prepared ligand recorded in d ^6^-DMSO as solvent showed the shifts (δ; ppm) of the protons affected by the chemical environment. The signal lying at high field side at (δ 3.847 ppm due to singlet ( S, 3 H ) of OCH_3_ group at para position of the benzene ring. No band broadness observed for protons of methoxy group because these protons not formed hydrogen bonds. These protons appeared as singlet band as this group has no neighbor’s electrons. The signal at high field 3.5 ppm signifying proton of phenolic OH group. Broadness caused by hydrogen bonding^28^. Multiple signals observed at range (7.1–7.5 ppm) because the aromatic protons of phenyl moiety appeared at different positions due to different environments around each proton. Signals at (δ 9.923–10.388 ppm) due to protons of aldehyde group^30^.

**Table 2:** Chemical shifts (δ; ppm) for protons of ligands.

(S,1 H) -OH	(m,5 H)-Ar-H	(S,3 H)-OCH_3_	(S,1 H)-CH = *N*	(S,1 H)-CH=O
3.5	7.200-7.432	3.847	9.771	10.4

**Fig. 4:**
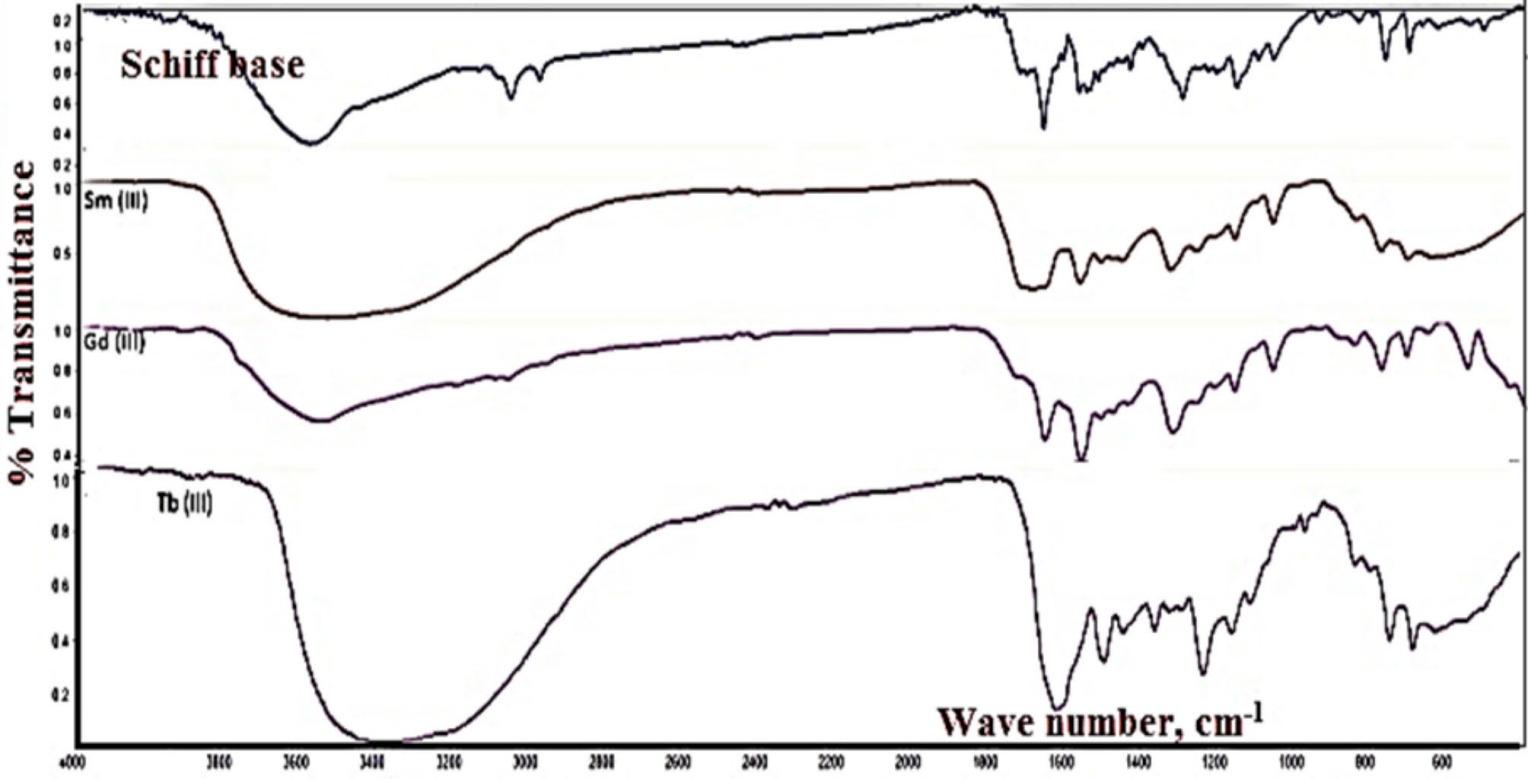
IR spectra of ligand and lanthanide complexes.

IR spectra, Fig. 4 confirmed bonding of ligand to lanthanide metal. Vibrational bands frequencies (cm^− 1^) listed in Table 3. Ligand vibrational frequencies of functional groups such as _C=N_, _C=O_ affected with different degrees depending on strength of π-interaction between the metal ion and free electrons on the function groups^29^. For the complexes: Broad band at frequency range (3345–3417 cm^−1^) assigned to OH stretch of the coordinated water molecules as confirmed from weight loss of thermal gravimetrical analysis. The medium intense band at 1600–1678 cm^−1^ of ligand (stretch C=O group) shifted to lower frequencies in complexes. This blue shift confirmed involvement of this functional group in metal complexation forming coordinate type bond with the metal ion. The medium-strong intense band at the frequency range 1510–1608 cm^−1^ assigned to stretching vibration of C=N also shifted to lower frequencies in the metal complexes. New bands at range (460–698 cm^−1^), (409–648 cm^−1^): M-O and M-N bonds respectively. Coordination bond formed between carbonyl ketone group of the ligand and the metal ion. Bonding mode between metal ion and ketone group of ligand via coordinate bond formation with the carbonyl group C=O group. Bonding mode of Cl^−^ ion and coordinating water molecules^29^.

**Table 3:** Characteristic IR frequencies (cm^− 1^) of ligand II and its complexes.

Sample	OHυ	δ OH _in plane_	_C=O_υ	_C=N(sy)_υ	_C=N(asy)_υ	_NH−Ph_υ	_M−O_υ	_M−N_υ
Ligand (L)	3425	1384	1600	1551	1253	1492	–	–
Sm L	3383	1283	1628	1509	1219	1458	693	634
Gd L	3396	1278	1596	1507	1124	1458	543	643
Tb L	3381	1331	1652	1499	1241	1450	692	634

The broad band at the frequency range 3345–3417 cm^− 1^ assigned to the OH stretching vibration of the coordinated water molecules. The presence of such water molecules assigned from molecular weight calculation and thermal analysis.

**Fig. 5:**
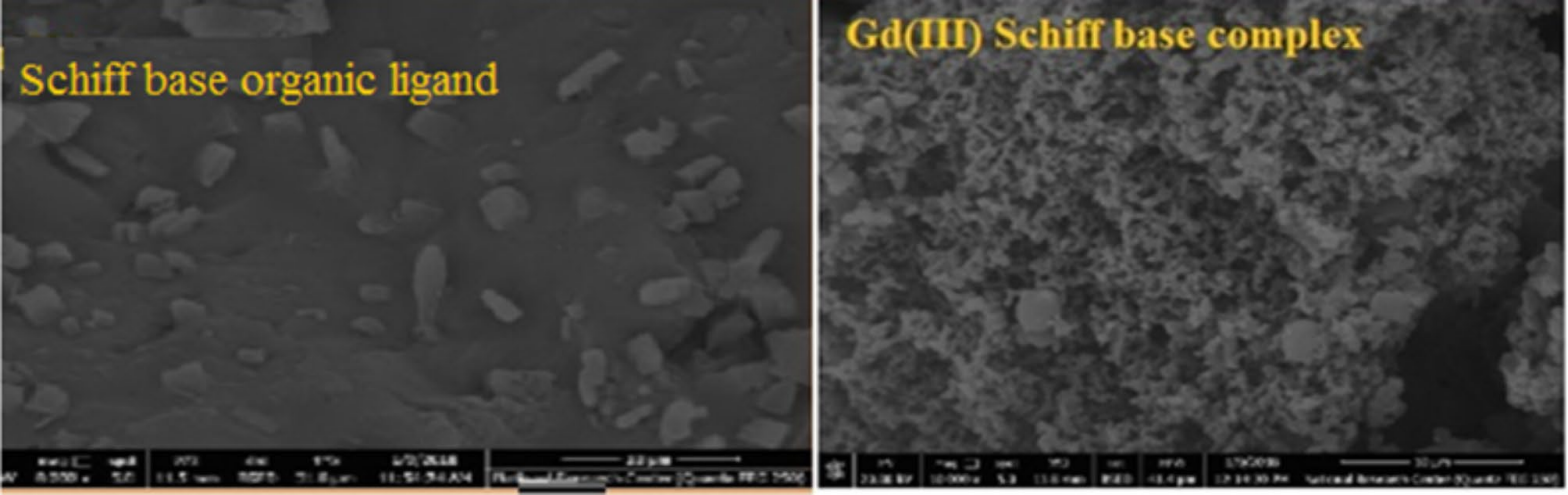
SEM micrographs of Schiff base ligand and gadolinium complex.

SEM micrographs revealed morphology of Schiff base at 10000x magnification, Fig. 5 Surface microstructure is changed from capsules, rude, crystalline and more appearance capsules shape in Schiff base ligand into reticulate, flower, cluster and crystalline structure respectively in the metal complexes^26^.

**Fig. 6:**
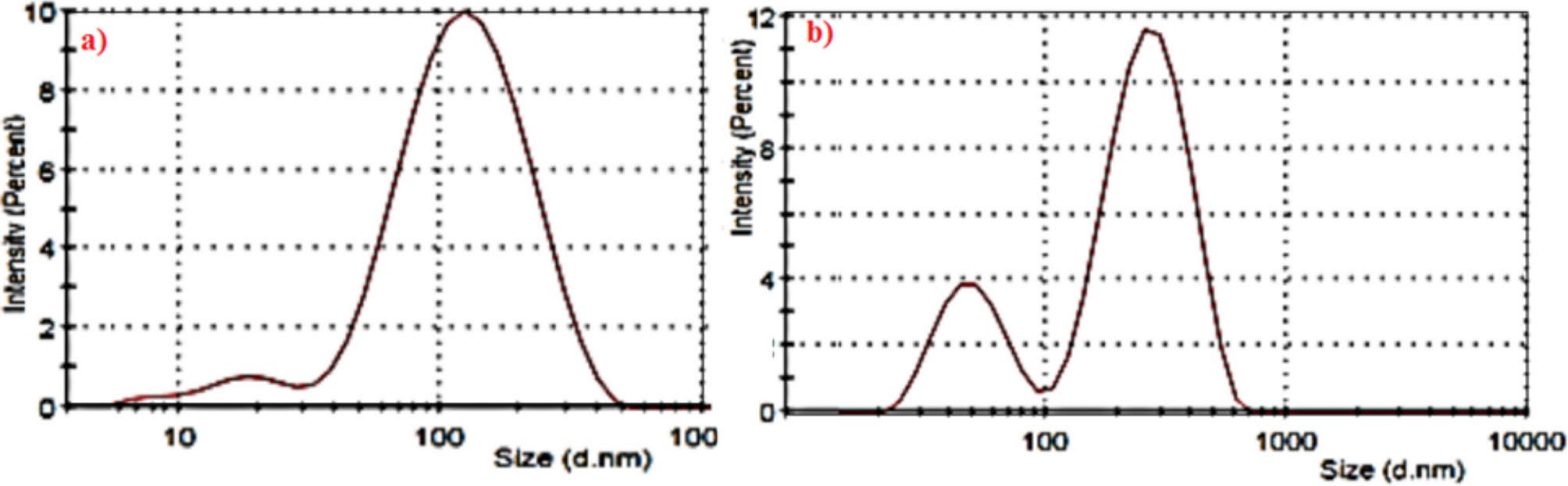
Particle size distribution: (**a**) The ligand, (**b**) Gadolinium complex.

The domains of particle size observed in morphology of gadolinium complex attributed to the particle size distribution. Figure 6 showed distribution of the average particle size of the ligand and the complex^30,31^.

The more intense peak of light scattering by colloidal particles of Gd complex confirmed the particle size domains observed in SEM micrographs. The morphology of Schiff base ligand changed on complexation. The rod like particles shape changed into continuous and self-assembled metal chelate via coordinate bond formation between the ligand and Gd(III) ion. These changes assessed by comparing particle size distribution curve of the ligand to that of the metal complex. The more intensities of scattered light are 12, 10 arbitrary unit for the metal complex and the ligand respectively confirmed the bond formation. The particle size distribution of the ligand Schiff base altered on binding the metal ion^30^.

### The optical activity of the metal complexes

**Fig. 7:**
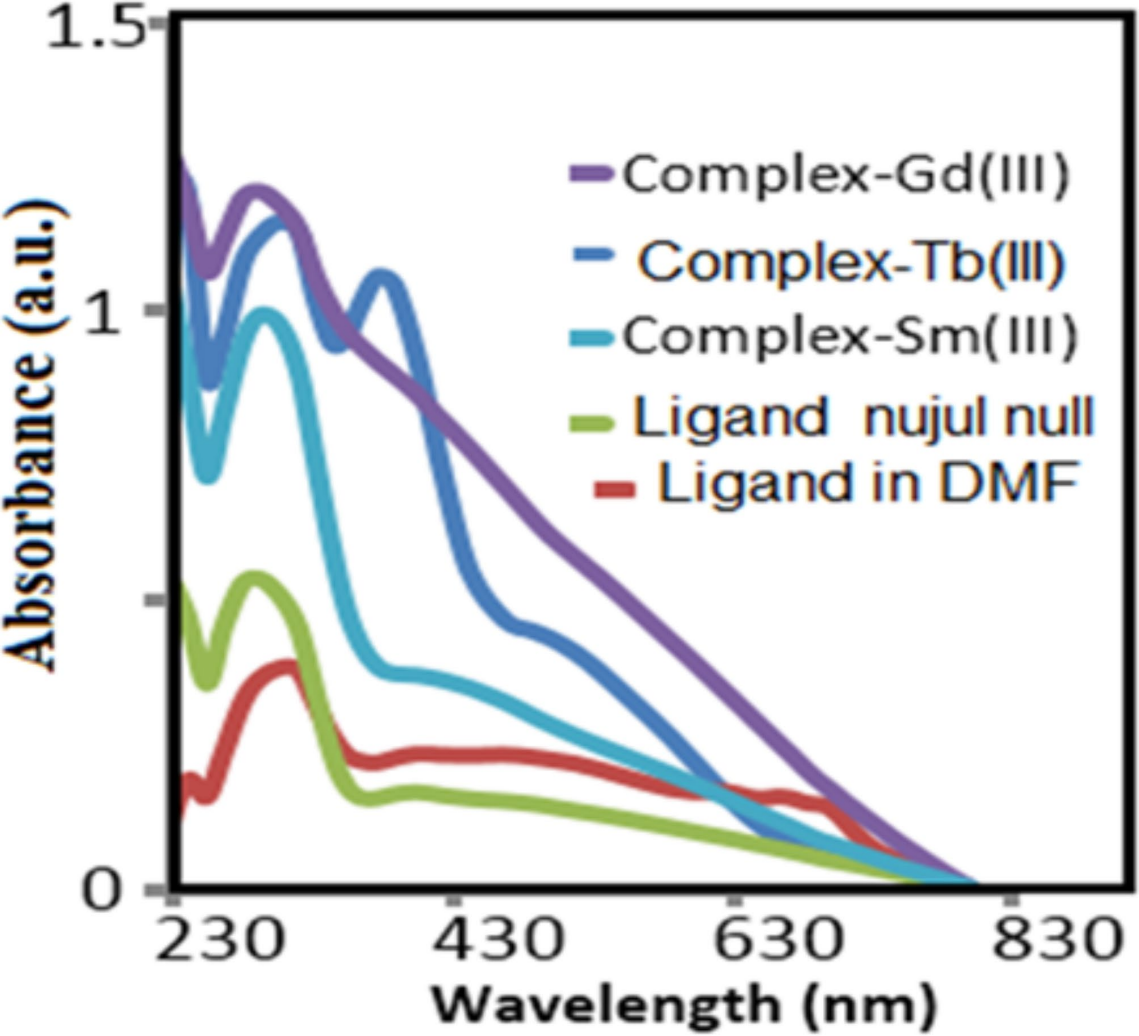
UV-Vis. spectra of Schiff base ligand and the metal complex.

The electronic absorbance spectra of the meal complexes shown in Fig. 7 in comparison to that of the free ligand (in Nujul mull and DMF respectively). The electronic spectral data listed in Table 4. Electronic spectra of Sm(III) complex exhibited absorption bands at 31,250 and 26,315 cm^− 1^ assigned to transitions ^6^H_5/2_ → ^4^F_9/2_, ^6^H_5/2_ → ^6^P_5/2_ and 6H5_/2_ → ^6^P_3/2_ respectively suggested octahedral geometry around Sm. Also Gd complex exhibited absorption bands at 31,230, 26,300 and 19,230 cm^− 1^ due to transitions ^4^I_9/2_ → ^4^P_1/2_, ^4^I_9/2_→^4^G_4/2_ and ^4^I_9/2_→^4^G_5/2_, ^2^G_5/2_ respectively confirmed octahedral geometry^27,31^.

Electronic transition by energetic UV- photon give intense absorption band especially for Gadolinium complex that gave the promising Schiff base complex based on the electronic configuration (1s^2^ 2s^2^p^6^ 3s^2^p^6^d^10^ 4s^2^p^6^d^10^f^7^ 5s^2^p^6^d^1^) contains one d-orbitals electron. The electronic configuration of the valence orbitals (4f^7^ 5d^1^ 6s^2^). The valence electrons in S, d easily excited giving the strong absorbance spectra). The absorbance wavelength of the metal complexes red-shifted by 30 nm more than ligand. High conjugated improved absorption UV-Vis. light. The intense optical absorbance of gadolinium complex confirmed rapid charge transfer. Terbium (atomic number 65 electrons are arranged in the electron configuration [Xe] 4f^9^ 6s^2^^27^.

The distinct absorption spectra Samarium and Gadolinium complexes reflected the different metal-ligand crystal field. For the same ligand, the spectra affected by the valence electrons in the conduction band of the metal complex. The electronic configuration of samarium 1s^2^ 2s^2^ 2p^6^ 3s^2^ 3p^6^ 3d^10^ 4s^2^ 4p^6^ 4d^10^ 4f^6^ 5s^2^ 5p^6^ 6s involved completely filled p orbitals. The electronic absorption bands in Gadolinium complex is more intense (absorbance 1.25 arbitrary units and red shifted up to 830 nm, so it more optically active and suitable for photodynamic therapy. At the same wavelength 240 nm, Sm complex showed much less intense band absorbance less than 1.0^32^.

**Table 4:** Electronic absorbance spectral data (cm^− 1^) and bonding parameters of oh complexes.

Complex	Metal complex chloride electronic spectral bands	Complex electronic spectral bands	Energy levels	Covalent parameter
Sm	37,037 33,333 30,030	31,250 26,315	^6^H_5/2_ → ^4^F_9/2_ → ^6^P_5/2_ → ^6^P_3/2_	β = 0.9921 b^1/2^ =0.0444 δ% = 0.796 η = 0.089
Gd	36,363 30,581 18,518	31,230 26,300 19,230	^4^I_9/2_ → ^4^P_1/2_ →^4^G_4/2_ →^4^G_5/2,_^2^G_5/2_	β = 0.9932 b^1/2^ =0.0412 δ% =0.684 η = 0.0827
Tb	37,037 33,333 30,030	29,411 23,809 19,230	^6^H_5/2_ → ^4^F_9/2_ → ^6^P_5/2_ → ^6^P_3/2_	β = 0.9991 b^1/2^ =0.044 δ% = 0.689 η = 0.0830

In UV electronic absorption spectra: the comparison of absorption bands between Sm(III) and Gd(III) is interesting. The red shift of the electronic absorption of Gd complex to higher wavelength up to 630 nm confirmed the photodynamic activity. The more favored in Gd complexes crystal ligand field stabilization energy decreased the splitting energy in the outer vacant d-orbitals and caused intense absorbance band compared to Sm complex (absorbance below 1.0^27^.

The complexes are good photosensitizers can be easily excited by low energy Vis. light photon. On relaxation to the ground state, the excited molecules donate the excited electrons to the inert cellular triplet oxygen rapidly generating various free radicals reactive oxygen species such as singlet oxygen ^1^O_2_, OH., superoxide (O_2_) ions oxidatively damage the components of the tumor cells including fatty acids, unsaturated cholesterol lipids, amino acid residues in proteins; alter bases of nucleic acids DNA & RNA; deactivating enzyme and the surrounding biomolecules including cell membrane. High yield and long of triplet state populated by intersystem crossing (ISC), increases the quantum yield of the singlet oxygen^31^.

The intensity of the absorbance bands changed by increasing the atomic number of the metal ion for the same chromospheres in the Schiff base ligand^18^. The fraction absorbance calculated using Eq. (1).

1 $${\text{Fraction}}\;{\text{absorbance}}\left( \alpha \right) = \frac{{2.3042 \times absorbance\;A}}{{{\text{sample}}\:{\text{thickness}}\:t}}$$

The energy gap (Eg) between highest occupied molecular orbital and the lowest occupied molecular orbital controlled UV-absorption coefficient and depends on the photon eneregy $\left( {h\nu } \right)$ according to Eq. (2).

2 $$\:\alpha\:h\nu\:=A{\left(h\upsilon\:-\text{E}\text{g}\:\right)}^{r}$$

**Fig. 8:**
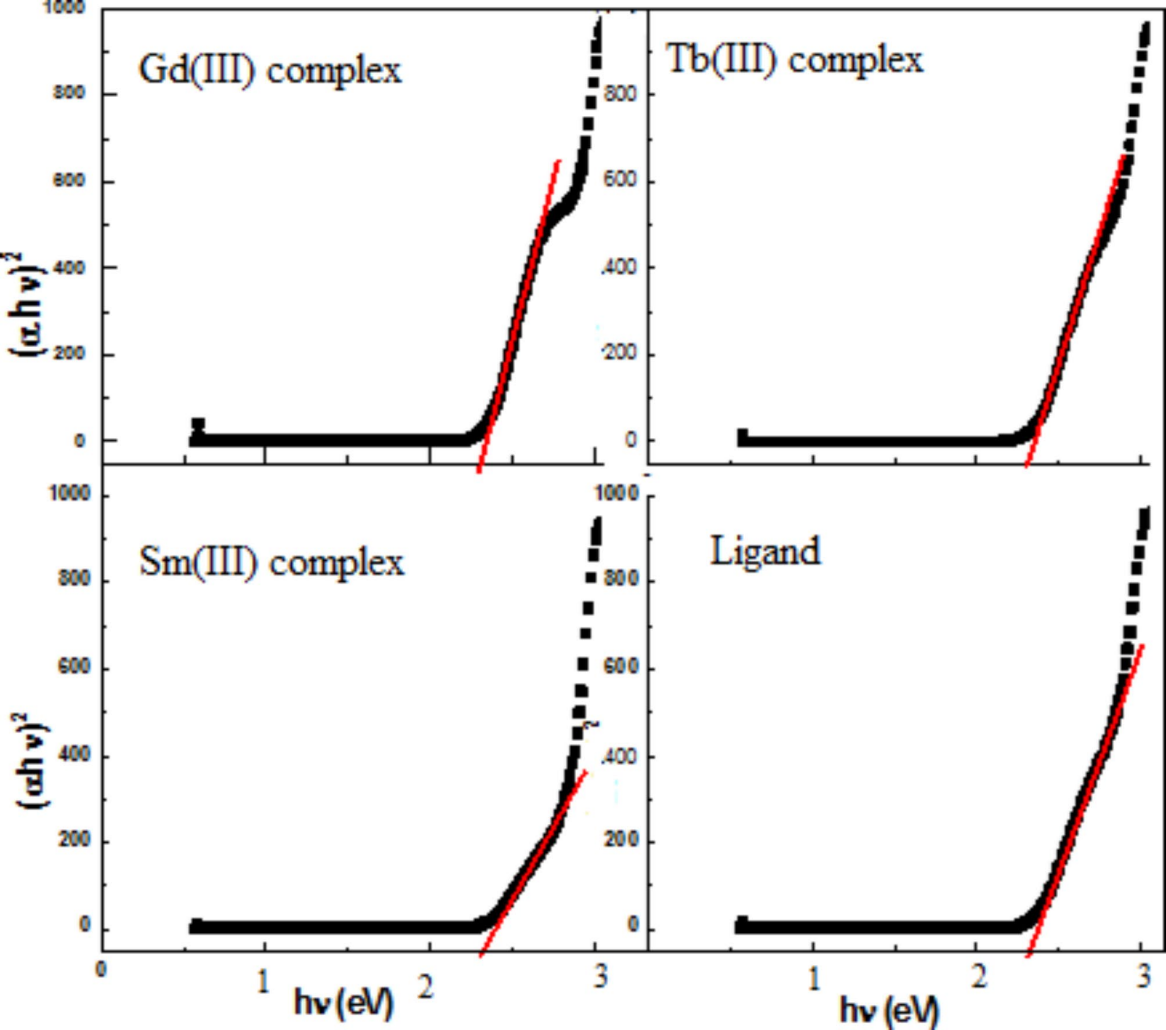
Tauc plots for metal complexes and ligand.

Where ν frequency of incident radiation is inversely proportional to absorbance wavelength (λ), A is a constant. The exponent r depends on nature of electronic transition. *r* = 2 for indirect transition, and r = ½ for allowed direct transition (only considered). Optical gaps, Eg from plot, Fig. 8. by interpolation the intersection of tangent line to x-axis^32,33^.

Low band gaps in the range (2.59–2.61 eV) confirmed good UV-absorbance of the metal complexes. The high optical band gap of the ligand (2.8 eV) decreased upon complexation. The electronic configuration specifically contributes to this change in transfer of lone pairs of electrons from the hetero atoms of the ligand to the vacant valence orbitals of the metal ions^33^. This finding confirmed by solving time dependent Schrodinger equation of metal complex at boundary conditions. The molecular modeling calculations of free ligand and complexes performed with Chem Bio Office 3D Ultra 11.0 using software installed on an IBM-compatible Pentium MMX-233 MHz personal computer. The optimized conformations (lowest energy) of the individual molecules determined using dynamic simulations followed by energy minimization to confirmed optical activity. The total energy (atomic unit) of the complexes followed the order:

$${\text{Sm }}( - 2401.1) > {\text{Tb }}( - 2533.7) > {\text{Gd }}( - 2666.3)$$

Low Eg indicated increase density of states available for occupation by electrons in regular octahedral crystal field^27^. Good optical properties of metal complexes confirmed by the high molar extension coefficient (ϵ) (M^− 1^cm^− 1^$\:\times\:{10}^{4}]$ for metal complexes:

$${\text{Gd (III) 13}}{\text{.55 > Tb(III) 13}}{\text{.4 > Sm(III) 12}}{\text{.6}}$$

Reflect good optical properties in comparison to various polyamides ($\epsilon$) 1.34 × 10^2^) prepared with tedious synthetic routs using toxic chemicals with moderate electronic absorbance^34,35^.

### Photo luminescence activity

**Fig. 9:**
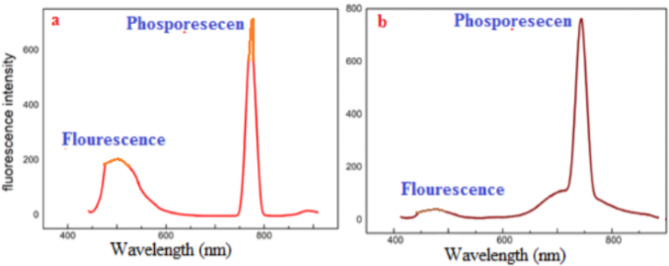
PL spectra: (**a**) Tb(III) and (**b**) Gd(III) complexes.

Figure 9 showed the comparative radiative photo tumescence activity of the optically active metal Gadolinium complexes.

The intense photoluminescence (phosphorescence band) recommended application in PDT. Intense fluorescence at 450 nm and 460 nm) and phosphorescence band at 710 nm confirmed photodynamic activity. Phosphorescence emission is more superior to fluorescence electronic transition in photodynamic therapy because it is slow photo physical process (long last continue after illumination). Phosphorescence radiation emitted by electronic transition populated by internal system crossing. Metal complexes showed less red shifted wavelength than hemato porphyrin fluorescence (in vivo red fluorescence. λ_max_.690 nm preferential uptake by neovasculature new blood vessels of tumor cells to allow sufficient time for conversion cellular triplet oxygen to singlet oxygen^33–35^.

Singlet oxygen apoptotic cancer cells by oxidative damage, macular degeneration, psoriasis, atherosclerosis, killing viruses, herbicides and insecticides. The protonated iminium group interact with biomolecules, via electron transfer form reactive excited singlet oxygen that form reactive oxygen species attack all the organic compounds in the tumor cells; It is very short-lived rapidly relaxes to ^3^O_2_ after affect biological systems^36,37^.

Long wavelength phosphorescence reflected: high rates of ISC and long life time of triplet state Hydrophilic hydrophobic characteristics by methoxy group of sensitizer metal complexes indicated solubility in biological media and absorption by cancer cells and diffusion to target site via blood stream. Low photo degradation enabled continuous production of singlet oxygen^29,30^ that kills cancer cells. Red-shifted λ absorption maxima chromophore improved tissue penetration. Sensitizers’ complexes could be rapidly absorbed by tumor cell to minimize radiation effect on patient^33–37^.

### Physicochemical properties

#### PXRD diffraction

**Fig. 10:**
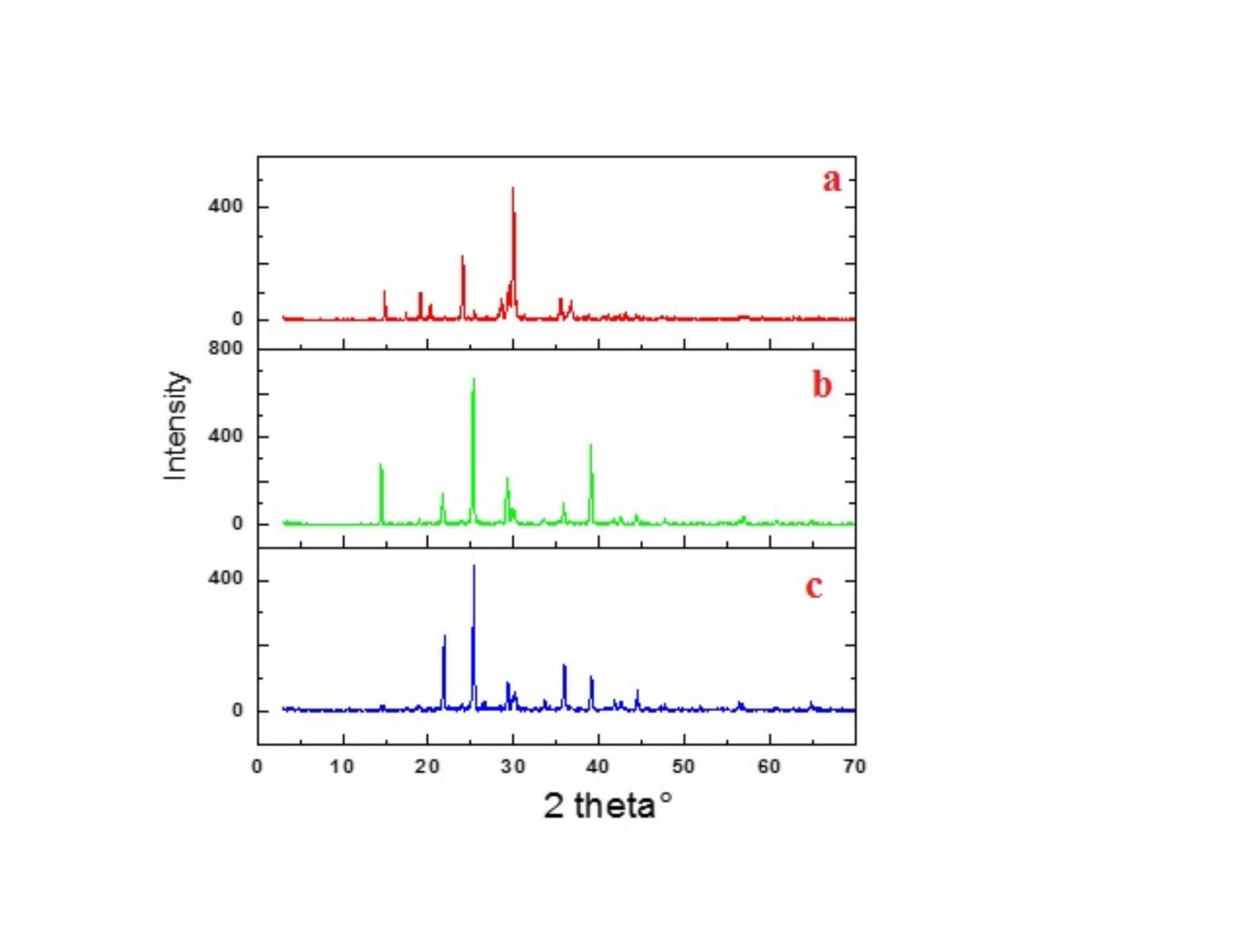
pXRD patterns: (**a**) Sm(III), (**b**) Gd(III) and (**c**) Tb(III) ion.

XRD patterns of metal complexes, Fig. 10 obeyed Bragg’s law represented by Eq. (3)^27,32^:

3 $${\text{n}}\lambda {\text{ = 2d sin}}\theta$$

where (θ) is the incidence and reflection angle, (d) is the distance between atomic planes, (λ) wavelength of incident X-ray beam, (n) is an integer number. All pXRD patterns showed prominent sharp diffraction peaks reflecting good crystal structure and lattice planes^27^.

Low intense peaks indicated the nanocrystalline strucutre. Such changes in the FWHM alter the particle size that calculated using Sherrer equation.The other microstructural constraints of metal complexs such as crystallite size (L), dislocation density (δ) and microstrain (ε) calculated using Eq. (4), collected in Table 5^27^:

4 $$L = \frac{{k\lambda }}{{\beta \cos \theta }},\delta = \frac{1}{{L^{2} }},\varepsilon \frac{{\beta \cot \theta }}{4}$$

**Table 5:** Microstructural parameter for metal complexes.

Metal complex	Particle size (L, nm)	Dislocation × 10^3^ (δ, nm^2^)	Strain ε × 10^3^
Sm(III)	12.54	6.36	6.90
Gd(III)	11.69	7.32	7.30
Tb(III)	10.59	8.92	8.04

The low dislocation density and residual microstrain reflected good purity of the prepared metal complexes^27^.

#### Thermal analysis

**Fig. 11:**
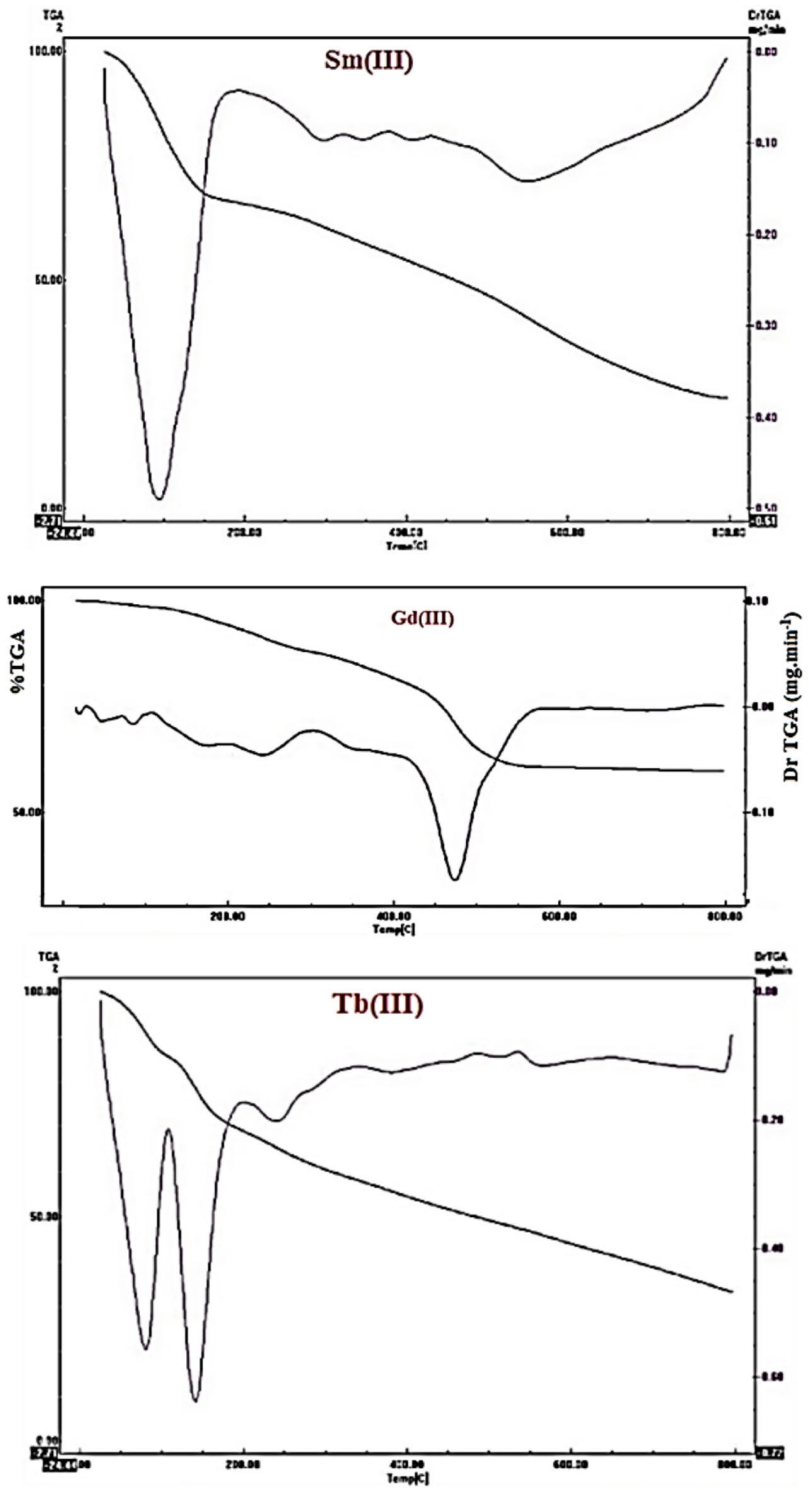
TGA-DTA curves of the prepared lanthanides metal complexes.

Complexes exhibit comparable thermal decomposition behavior represented in Fig. 11. The TGA curves of the metal complexes showed rapid thermal decomposition at the first step below 100 °C confirmed the presence of coordinated water molecules as a ligand in the inner sphere of the metal complexes. Remaining decomposition steps attributed to the decomposition of the organic parts of the metal complexes leaving the metal oxides residues^27,32^.

TG-DTA curves showed thermal degradation by dehydration, decomposition and formation metal oxide residue. TGA involved mass loss on heating sample at a predetermined rate and preferably linear rate. Samarium complex showed decompositions: 25–199.31 °C: mass loss 33% (cal.32.34%) with endothermic peak with t_min_ 94.2 °C due to removal lattice water molecules and HCl; 199.31–413.24 °C, wt.loss 13% (cal.12.83%) due to removal coordinated water molecules give an endothermic peak at 409.56 °C, 413.24–659.8 °C wt.loss 20% (cal.20.6%) corresponds to decomposition organic part of ligand^34^. Mass loss at 659.8–799.06 °C: mass loss 8% (cal.8.19%) due to further ligand decomposition and oxide formation.

Gadolinium complex showed decomposing steps: 25–125 °C, wt.loss 2.664% (cal. 3.092%) with exothermic peak at 107.32 °C due to removal of lattice water; 125–275.85 °C, mass loss 9.27% (calc.9.27%) due to removal coordinated water. Endothermic peak at 241.23 °C; 275.85–565 °C, mass loss 29.3% (cal.30.9%) corresponds to decomposition organic part of ligand. Oxide formation showed endothermic minimum at 474.34 °C.

TG curve of Tb(III) complex: 25–96.87 °C, mass loss 12.17% (cal.12.13%) accompanied by endothermic peak at 81.24 °C signified removal lattice water molecule and HCl; 96.87–187.7 °C, mass loss 17.39% (calcd.18.1%) due to removal coordinated water molecules and HCl. Endothermic peak at 141.33 °C, 187.7–424.31 °C: wt.loss 17.39% (cal.19.4%) corresponds to decomposition organic part of ligand, endothermic peak at 240.56 °C: 424.31 to 798.99 °C: mass loss 19.12% (calculated 0.20.08%) due to further decomposition of ligand and metal oxide formation. Table 6 represented the assigned decomposition products.

**Table 6:** Thermal analysis gadolinium complex.

Chemical formula (Mw.)	TG range (^°^C)	Mass loss %		Assignment		%Metal oxide
		Cal.	Found			
C_16_H_16_N_2_O_4_Gd.2H_2_O.2Cl	25–125	3.09	2.66	Dehydration lattice water		12.65
	125–276	9.27	9.3	Loss coordinated water		
	276–565	30.90	29.3	Loss organic ligand		

Structural properties of complexes depends of the type of the metal ion, Kinetics parameters of thermal decomposition (E_a_) and n determined from TGA and DTG following various models considering special features of decomposition mechanisms using Eqs. (5–13)^38^.

5 $${\text{Degree}}\:{\text{decomposed}},\alpha \:\:\: = \frac{{{\text{w}}_{ \circ } - {\text{w}}_{{\text{t}}} }}{{{\text{w}}_{ \circ } - {\text{w}}_{\infty } }}$$

where w, w_t_ and w_∞_ are sample wt. loss before degradation, at time t and after decomposition, respectively.

6 $${\text{Reaction}}\:{\text{rate}}\:\left( {\frac{{{\text{d}}\upalpha }}{{{\text{dt}}}}} \right) = {\text{K}}\left( {\text{T}} \right){\text{g}}\left( \upalpha \right)$$

where t is the time, T: absolute temperature, K(T) is temperature dependent function and g(α) is kinetic conversion function. Arrhenius equation represented by Eq. 7.

7 $${\text{Rate}}\:{\text{constant}}\:{\text{k}} = {\text{A}}\;{\text{exp}}\left( { - \frac{{{\text{E}}_{{\text{a}}} }}{{{\text{RT}}}}} \right)$$

where A is pre-exponential factor (kJ mol^− 1^) indicates reaction speed, R is universal gas constant J mol^−1^ K.

8 $$\frac{{{\text{d}}\alpha }}{{{\text{dt}}}} = {\text{A}}\;{\text{exp}}\left( { - \frac{{{\text{E}}_{{\text{a}}} }}{{{\text{RT}}}}} \right){\text{g}}\left( \alpha \right)$$

Thermal decomposition reaction carried out under a linear temperature program (T = T_o_ + βt).

Where heating rate β = dT/dt, T_o_: starting temperature assuming 1^o^ order (*n* = 1) degradation.

9 $${\text{ln}}\left( {1 - \upalpha } \right) = - \frac{{\text{A}}}{\upbeta }\int\limits_{{{\text{T}}^{ \circ } }}^{{\text{T}}} {{\text{Exp}}\left( { - \frac{{{\text{E}}_{{\text{a}}} }}{{{\text{RT}}}}} \right){\text{dT}}}$$

10 $${\text{ln}}\left( {\frac{{{\text{g}}\left( \upalpha \right)}}{{{\text{T}}^{2} }}} \right) = {\text{ln}}\left\{ {\frac{{{\text{AR}}}}{{\upbeta {\text{E}}_{{\text{a}}} }}\left( {1 - \frac{{2{\text{RT}}}}{{{\text{E}}_{{\text{a}}} }}} \right)} \right\} - \frac{{{\text{E}}_{{\text{a}}} }}{{{\text{RT}}}} = {\text{ln}}\frac{{{\text{AR}}}}{{\upbeta {\text{E}}_{{\text{a}}} }} - \frac{{{\text{E}}_{{\text{a}}} }}{{{\text{RT}}}}$$

where g(α) = 1 − (1 − α)^1−n^ /1 − n for *n* ≠ 1 and g(α) = -ln(1 − α) for *n* = 1. Correlation coefficient (r) computed using least squares method for n: 0–1, Table 7.

Plot $\:\text{ln}\left(\frac{\text{g}\left({\upalpha\:}\right)}{{\text{T}}^{2}}\right)-$1/T gave the order at the best fit (*r* ≈ 1) for decomposition state of interest. Ea, A calculated from slope and intercept.

Activation parameters of decomposition calculated using Eqs. (11–13) informed about thermal decomposition mechanism^36^:

11 $${\text{Enthalpy}}\left( {\Delta {\text{H}}^{{\text{*}}} } \right) = {\text{E}}_{{\text{a}}} - {\text{RT}}$$

12 $${\text{Entropy}}(\Delta S^{*} ) = R\ln \frac{{hA}}{{K_{B} T}}$$

13 $${\text{Free}}\:{\text{energy}}\:(\Delta {\text{G}}^{{\text{*}}} ) = \Delta {\text{H}}^{{\text{*}}} - {\text{T}}\Delta {\text{S}}^{{\text{*}}}$$

**Fig. 12:**
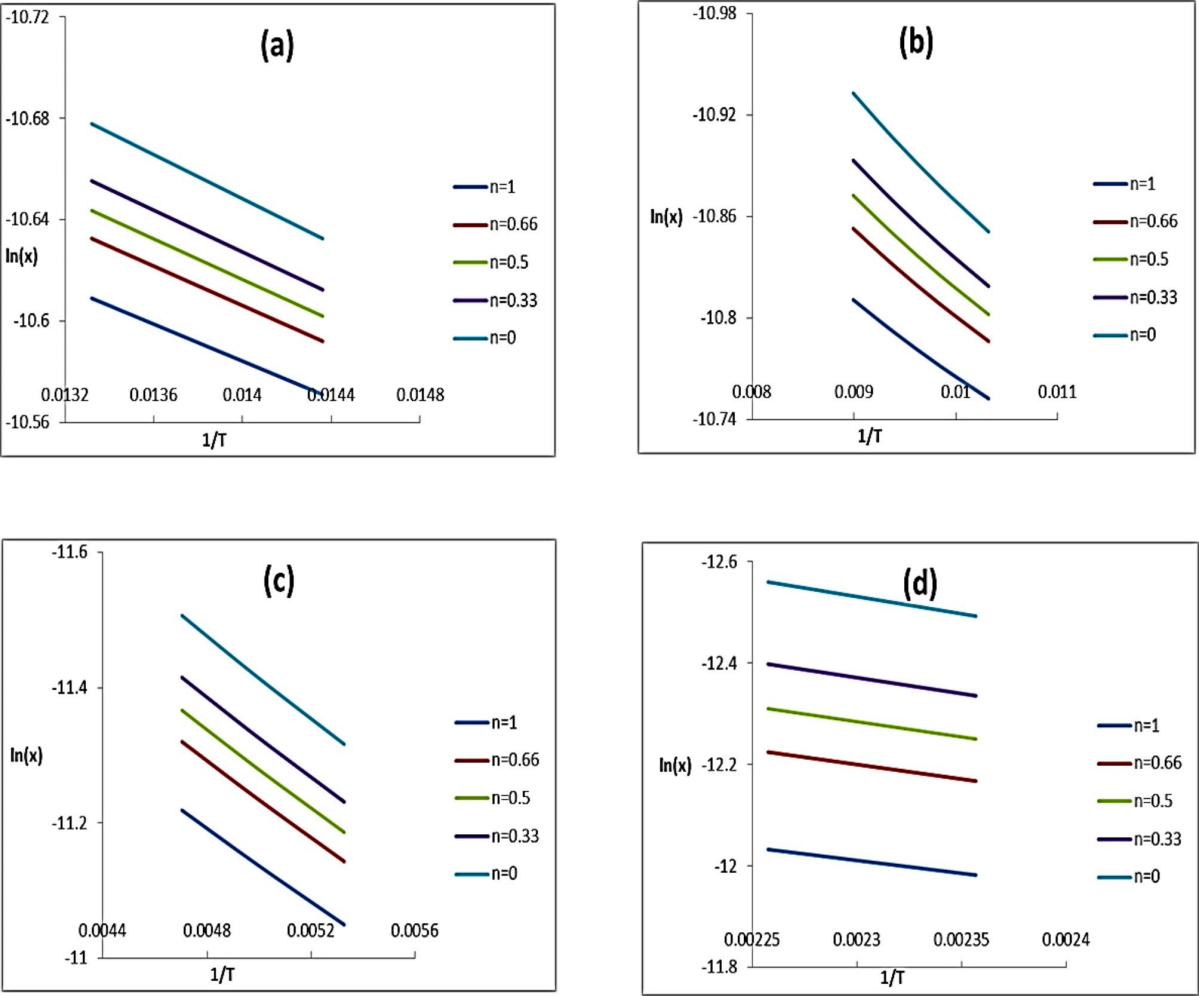
Coats-Redfern plots: (**a**) first, (**b**) second, (**c**) third and (**d**) fourth degradation steps.

Where ∆H* and ∆G* (kJ mol^− 1^) entropy (ΔS^*^) kJ mol^− 1^K^− 1^, h plank constant and $\:{\text{K}}_{\text{B}}$ Boltzmann constant. Linear plots, Fig. 12 confirmed 1^o^ kinetics for decomposition reactions of all steps showed a best fit for *n* = 1. Table 8 showed values Ea, ΔG^*^ of confirmed thermal stability of metal complexes. E_a_ decomposition increased on successive decomposition subsequent degradation steps revealing thermally stable remaining part^38^.

**Table 7:** Parameters of linear fitting of thermal decomposition data at mid temperature.

T.°K	n	r		T.°K	n	r	T.°K	n	r
344	0	0.993		473	0	0.998	693	0	0.982 0.9948
	0.33	0.9931			0.33	0.9988		0.33	
	0.5	0.9932			0.5	0.999		0.5	0.9971
	0.66	0.9934			0.66	0.9998		0.66	0.9983
	1	0.9935			1	1		1	0.9995

The low value Ea of decomposition indicated auto catalyzed thermal decomposition by the metal ion. The value of activation energy and the thermal stability of the metal complexes followed the order.

$${\text{Gadolinium > Terbium > Samarium}}$$

Complex Sm(III) and Tb(III) complexes had high thermal stability by covalent bonding Positive ΔS^*^ indicated activated complexes more ordered than reactant in these slow reactions. The Orderings due to polarization of bonds in activated state through electron transfer. Negative ΔH^*^ explained exothermic decomposition process. Positive ΔG^*^ indicating slow nonspontaneous decomposition reaction; increased significantly for subsequent decomposition stages. Increasing$\:\:\:\text{T}\varDelta\:{\text{S}}^{\text{*}},\:$reflected low rate of removal subsequent species^38^.

**Table 8:** Kinetic parameters for thermal decomposition complexes.

Complex	Step	Ea	A x10^− 5^	− ΔH^*^	ΔS^*^	ΔG^*^
Sm(III)	1	0.81	1.30	2.40	0.29	110.05
	2	2.80	1.05	2.01	0.30	169.63
	3	3.56	0.97	3.18	0.30	239.74
	4	7.15	0.95	1.18	0.30	301.30
Gd(III)	1	0.24	0.42	2.62	0.30	100.53
	2	0.89	0.57	3.04	0.30	138.82
	3	1.80	3.07	3.96	0.29	196.35
Tb(III)	1	0.30	0.83	2.47	0.29	95.67
	2	0.41	1.00	3.04	0.29	119.02
	3	2.25	1.52	2.56	0.29	167.31
	4	4.23	1.44	3.12	0.30	259.76

## Conclusion

The new prepared ligand Schiff base ligand 4-methoxy salicaldhyde (3-hydrazonobutan-2-one) coordinated metal ions. Electron withdrawing methoxy group prevented aggregation and improved both solubility and stat ability. The ligand contains prepared contains many chromosphere that intensified and red shifted electronic absorbance of the metal complexes. Metals complexes are good photosensitizer as confirmed by the intense phosphorescence band. The ligand binding the lanthanide metal ions Sm(III), Gd(III) and Tb(III) by coordinate bond formation. Intense absorption bands λ_max_.280–390 nm and intense phosphorescence bands up to 730 nm of these metal complex confirmed photo dynamic activity and ability to interact with molecular triplet oxygen giving reactive oxygen species that destroy cancer cells. Low charge transfer energy: 2.59–2.61 eV and high molar extinction coefficients ε $\:\:6\times\:{10}^{4}$ M^− 1^ cm^− 1^ reflected good optical activity satisfied application in PDT.

## Data Availability

All data generated or analysed during this study are included in this published article.
